# Non‐inferiority analysis of a fetal heart rate artificial intelligence algorithm to registered nurse assessment

**DOI:** 10.1002/pmf2.70433

**Published:** 2026-08-27

**Authors:** Rohit Pardasani, Renee Vitullo, Sara Harris, Karen P. Becker, Halit O. Yapici, John W. Beard

**Affiliations:** ^1^ Patient Care Solutions GE HealthCare Chicago Illinois USA; ^2^ Boston Strategic Partners Inc Boston Massachusetts USA

**Keywords:** artificial intelligence, cardiotocography, clinical decision‐making, fetal monitoring, non‐inferiority

## Abstract

**Introduction:**

Cardiotocography (CTG) is widely used for monitoring fetal heart rate (FHR) and uterine activity (UA) during pregnancy and labor. Clinician interpretation of CTG tracings may be subjective, leading to significant inter‐ and intra‐observer variability and inconsistent clinical decision‐making. An artificial intelligence (AI) algorithm using deep learning and rule‐based techniques was developed to label FHR and UA data during labor. Further development of such algorithms may add future objective monitor outputs to support clinician interpretation. This study assessed the non‐inferiority of the algorithm's performance compared to registered nurse (RN) reader assessment.

**Methods:**

This retrospective non‐inferiority assessment utilized CTG data across five US hospital facilities (2013–2024). CTG labeling assessment by the AI algorithm was compared with that of RN readers for primary endpoints (baseline value, accelerations, decelerations, contraction frequency, and contraction duration) and secondary endpoints (variability and deceleration types), using a panel of qualified expert RNs who accepted or rejected the assessment. Exploratory endpoints included frequency of true and false positives/negatives and sensitivity/specificity of accelerations and decelerations. A non‐inferiority threshold of 15% was required between the RN reader and algorithm.

**Results:**

Of 2639 de‐identified fetal tracings, 800 were randomly selected for this study. The most frequently reported maternal characteristics were full‐term gestational age (*n* = 355; 44.38%) and non‐Hispanic White (*n* = 409; 51.13%). The algorithm performed comparably to the RN reader for all endpoints except contraction duration, with percent differences (95% confidence interval [CI]) in acceptance rates (ARs) of 3.38% (−1.5%, 8.25%) for baseline value, 3.03% (0.72%, 5.34%) for acceleration, 7.14% (3.68%, 10.61%) for deceleration, 8.25% (3.57%, 12.93%) for contraction frequency, 22.13% (17.39%, 26.86%) for contraction duration, −1.75% (−4.10%, 0.60%) for variability, and 9.13% (5.38%, 12.88%) for deceleration type. For contraction duration, AR decreased to 13.75% (9.89%, 17.61%) when allowing for a 20‐s range of variability to account for visual approximations.

**Conclusion:**

This study demonstrated the non‐inferiority of a novel AI algorithm compared to RN readers in analyzing and assessing fetal tracings. These findings highlight AI's potential to serve as a decision‐making support tool in obstetric care. By providing consistent analyses, the algorithm may help reduce observer variability and enhance clinicians’ confidence in identifying fetal parameters.

## 1 INTRODUCTION

Cardiotocography (CTG) is used in obstetric care to monitor fetal well‐being during pregnancy and labor [1]. By simultaneously measuring and recording fetal heart rate (FHR) and uterine activity (UA), CTG offers insights into the physiological state of the fetus and its response to the intrauterine environment. The interpretation of CTG tracings plays a critical role in identifying changes in fetal well‐being, such as hypoxia, and guiding timely clinical interventions to optimize maternal and neonatal outcomes [2].

Although a widely used tool in obstetrics and gynecology, CTG interpretation has been inconsistent amongst clinicians [3]. Clinicians analyze various parameters of FHR and UA, including baseline rate, variability, accelerations, decelerations, and contraction patterns, to assess fetal condition. These interpretations largely depend on clinicians’ expertise, and their subjective nature has led to significant inter‐ and intra‐observer variability, posing challenges to consistent clinical decision‐making [4]. Improvement of CTG interpretation could reduce unnecessary variability in care and optimize medical interventions, leading to improved health outcomes for the mother and fetus [5]. The National Institute of Child Health and Human Development (NICHD) developed guidelines to standardize CTG interpretations, provide clear definitions and classifications of FHR and UA, and serve as a foundation for integrating emerging technologies to enhance the objectivity and predictive value of CTG [3]. The American College of Obstetrics and Gynecology has also recently addressed intrapartum FHR interpretation and management in an updated practice guideline. These reference documents have addressed many, but not all, of the inconsistencies in the field of CTG monitoring [6].

Deep learning and artificial intelligence (AI) methods have been proposed to address clinician CTG interpretation variability [1]. By using rule‐based analysis and AI algorithms, the subjectivity in assessing FHR and uterine contraction patterns can be reduced, and clinicians can monitor fetal well‐being more consistently [7]. An FHR AI algorithm was previously developed using deep learning and rule‐based techniques to analyze and assess FHR and UA parameters during labor [8]. The algorithm was trained by supervised learning from labeling done by qualified registered nurses (RNs) based on the NICHD guidelines to calculate the FHR baseline and describe events, related parameters (acceleration, deceleration, variability, tachycardia, and bradycardia), contractions (frequency, duration, peak, resting tone, Montevideo units, and tachysystole presence), and tracing assessment.

This study analyzed the FHR AI algorithm's performance against RN readers’ CTG assessments. We hypothesized that the FHR AI performance would not be inferior to that of RN readers, as measured by the acceptance rate (AR) of CTG assessments by an RN review panel.

## 2 MATERIALS AND METHODS

### 2.1 Datasets and sample size

The data were sourced by HCA Healthcare, Inc. and included demographic information (e.g., age, gestational age, race, and body mass index [BMI]) as well as CTG monitoring data acquired using varying maternal‐fetal monitor types and manufacturers in the United States between 2013 and 2024. This retrospective study was determined exempt from Institutional Review Board (IRB) oversight since the research and analysis were limited to data gathered initially for non‐research purposes, adhering to privacy laws and IRB exemption criteria 45CFR46.104(d)(4). In accordance with national legislation and institutional requirements, informed consent was not required for the use of this de‐identified, retrospectively collected data. All research activities were conducted in accordance with the ethical principles of Good Clinical Practice and applicable regulations originating from the Declaration of Helsinki. Records were excluded if they contained < 30 min of FHR data. One 30‐min fetal tracing was included from each admission. A power calculation was performed with conservative assumptions (α level: 5%; Power: 85%), which determined a sufficient sample size of 731 fetal tracings to assess non‐inferiority. Tracing selection was performed using stratified random sampling to ensure balanced representation across key subgroups, including site, race and ethnicity, maternal age, estimated gestational age, and BMI. To minimize selection bias, samples were weighted within strata, and tracings from underrepresented subgroups were included in full.

### 2.2 RN participants

RNs were recruited to serve as “RN readers” or “panel members” based on their current role, years of experience in labor and delivery, certifications, and prior fetal monitoring training. Specifically, RN readers were registered nurses in obstetrics and gynecology trained in the NICHD guidelines, with ≥5 years of experience as practicing RNs in a labor and delivery or obstetrics and gynecology department. RN panel members had the same qualifications as RN readers but also held a Certified Electronic Fetal Monitoring subspecialty certification. A total of 10 RN readers and 9 RN panelists participated in this study.

### 2.3 Non‐inferiority assessments

The algorithm was required to achieve a 15% non‐inferiority margin compared to RN readers to evaluate the performance of software device and derived functions (Table 1). The non‐inferiority threshold of 15% was determined based on a reference study that contained an accuracy lower bound (95% confidence interval [CI]) of at least 85% when benchmarked against FHR baselines documented by experts [9]. Non‐inferiority was measured as the difference in AR between the RN readers and the algorithm (H_0_: [AR_RN reader_] − [AR_algorithm_] ≥ 0.15; H_1_: [AR_RN reader_] − [AR_algorithm_] < 0.15). Differences in AR were estimated using generalized estimating equations with a binomial distribution and identity link function to account for the paired study design. For the descriptive exploratory subgroup analyses, the same methodology was utilized. Non‐inferiority was established upon rejecting the null hypothesis and obtaining a *p* value < 0.05. Specifically, these tests were based on a two‐sided 95% CI, in which non‐inferiority was established if the upper limit of the 95% CI (calculated using robust variance estimators) was < 15%.

**TABLE 1:** Overview of the algorithm labeling functions.

Functions	Assessment outputs
Software device	
Baseline	FHR Baseline value (in bpm) rounded to nearest 5 bpm or No Baseline
Acceleration	Present or absent
Deceleration	Present or absent
Contraction Frequency	Low‐high rounded to nearest 0.5 min
Contraction Duration	Low‐high rounded to nearest 10 s
Derived	
Variability	Absent, minimal, moderate, marked, or none
Deceleration Types	*Multi‐select*: Late, Variable, Early, Undefined, Prolonged, or None

Abbreviations: bpm, beats per minute; FHR, fetal heart rate.

### 2.4 Procedures

All fetal tracings were independently labeled by the AI algorithm and RN readers, both of whom assessed various parameters, including software device functions (FHR baseline, accelerations, decelerations, contraction frequency, and contraction duration), as well as derived functions (variability and deceleration types) (Table 1). For each individual intrapartum tracing, the labeled assessments from the algorithm and RN readers were paired as a set of two and randomized into three review blocks. Each block was randomly assigned to one of the three panels, each of which consisted of three members who reviewed the assessments side by side in virtual meetings. Panelists were blinded to whether the assessment was from the AI algorithm or RN reader. For each set of paired tracing parameter assessments, the panel discussed any discrepancies and used NICHD definitions to reach a final consensus on whether to accept one, both, or neither, and the ARs were calculated as percentages. For instance, panelists utilized NICHD guidelines for acceptance criteria and allowed for a ± 5 bpm baseline variance [3].

The primary endpoints for the study were the software device functions (baseline value, accelerations, decelerations, contraction frequency, and contraction duration). The secondary endpoints included the derived functions (variability and deceleration types), which were tested under the same non‐inferiority margin. A confusion matrix and calculations of sensitivity and specificity for the acceleration and deceleration functions were also assessed as exploratory endpoints. The confusion matrix detailed the frequency of tracings that were true positives, false positives, true negatives, and false negatives based on panel acceptance, while sensitivity and specificity were derived from those metrics. Lastly, exploratory demographic subgroups (race and ethnicity, BMI, and site) were analyzed descriptively.

### 2.5 Reanalysis of contraction duration

**FIGURE 1:**
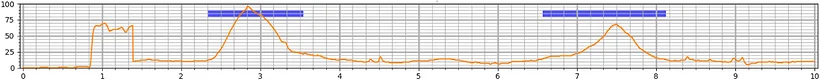
Sample uterine activity waveform tracing. A sample uterine activity waveform that requires visual approximation to assess contraction duration onset and offset, leading to subjectivity. The adjusted window factors in an uncertainty of up to one subdivision (± 10 s/one grid box) can reasonably occur at both the start and end markers.

A post hoc analysis of contraction duration was conducted using a ±20‐s tolerance window to account for the inherent subjectivity of contraction onset and offset. On standard 10‐min fetal monitor grids, the smallest visual subdivisions represent 10‐s intervals (Figure 1). Because visual approximations can vary by about one small grid box (±10 s) at both the start and end of the contraction, the cumulative expected visual variance accounts for ±20 s. Following the ±20‐s adjustment, the panel response was retrospectively classified as “Both” if the panel accepted either the FHR AI or RN reader interpretation and the actual difference between the minimum and maximum durations was < 20 s.

To further validate this window, a second post hoc analysis was conducted to evaluate the frequency distribution of differences in paired contraction duration values. Tracings where the panel responded ‘None’ were excluded because neither assessment was deemed clinically acceptable. The adjusted analysis was performed on the remaining 470 tracings. The minimum and maximum duration values per tracing were treated as independent observations (*n* = 940) to calculate the mean and median differences in contraction duration.

## 3 RESULTS

**FIGURE 2:**
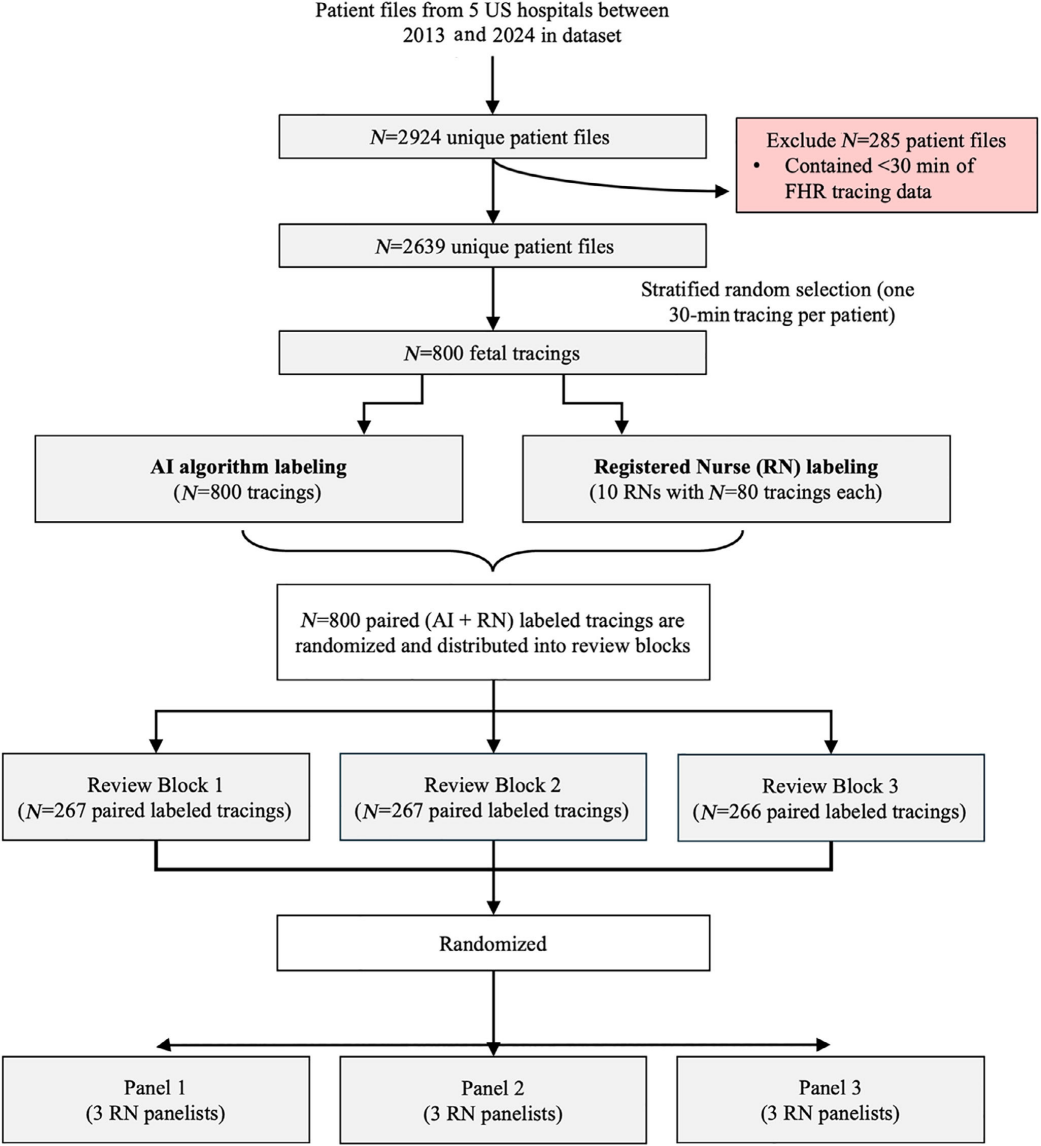
Study design. This flowchart details the study design, outlining the data inclusion and multistage comparative labeling process between an AI algorithm and RN readers. AI, artificial intelligence; FHR, fetal heart rate; RN, registered nurse.

The dataset yielded 2924 unique patient files during the study period. A total of 285 patient files were excluded for <30 min of FHR tracing data, resulting in 2639 patient files included in the study. Based on the sample size calculation and adjusting conservatively for any uninterpretable data during the tracing annotation phase, a final sample of 800 fetal tracings was selected for inclusion (Figure 2). The 800 tracings were then provided to the AI algorithm and the 10 RN readers (80 tracings per RN reader) for assessment and labeling.

The maternal characteristics of the final dataset are provided in Table 2. The most frequently reported estimated gestational ages were full‐term (*n* = 355; 44.38%), followed by early‐term (*n* = 222; 27.75%), and preterm (*n* = 93; 11.63%). The most represented ethnicities and races were non‐Hispanic White (*n* = 409; 51.13%), non‐Hispanic Black (*n* = 130; 16.25%), and Hispanic (*n* = 117; 14.63%).

**TABLE 2:** Study sample demographics.

Demographic variable	Tracings, *n* (%) *N* = 800
Race and ethnicity	
Non‐Hispanic White	409 (51.13%)
Non‐Hispanic Black	130 (16.25%)
Hispanic	117 (14.63%)
Non‐Hispanic Asian	66 (8.25%)
Other Race or Ethnicity	76 (9.50%)
Missing	2 (0.25%)
Age (in years)	
Under 20	30 (3.75%)
20–29	282 (35.25%)
30–39	444 (55.50%)
40 or above	44 (5.50%)
BMI	
<18.5	6 (0.75%)
18.5 to <25	164 (20.50%)
25 to <30	223 (27.88%)
≥30	375 (46.88%)
Missing	32 (4.00%)
Estimated gestational age—in weeks	
Preterm (<37)	93 (11.63%)
Early term (37 to <39)	222 (27.75%)
Full term (39 to <41)	355 (44.38%)
Late term (41 to <42)	43 (5.38%)
Post term (≥42)	9 (1.13%)
Missing	78 (9.75%)
Site	
1	456 (57.00%)
2	223 (27.88%)
3	75 (9.38%)
4	30 (3.75%)
5	16 (2.00%)

*Note*: “Other Race or Ethnicity” per hospital records includes Islam, Nepali; Not Hispanic or Latino (in ethnicity).

Abbreviation: BMI, body mass index.

**FIGURE 3:**
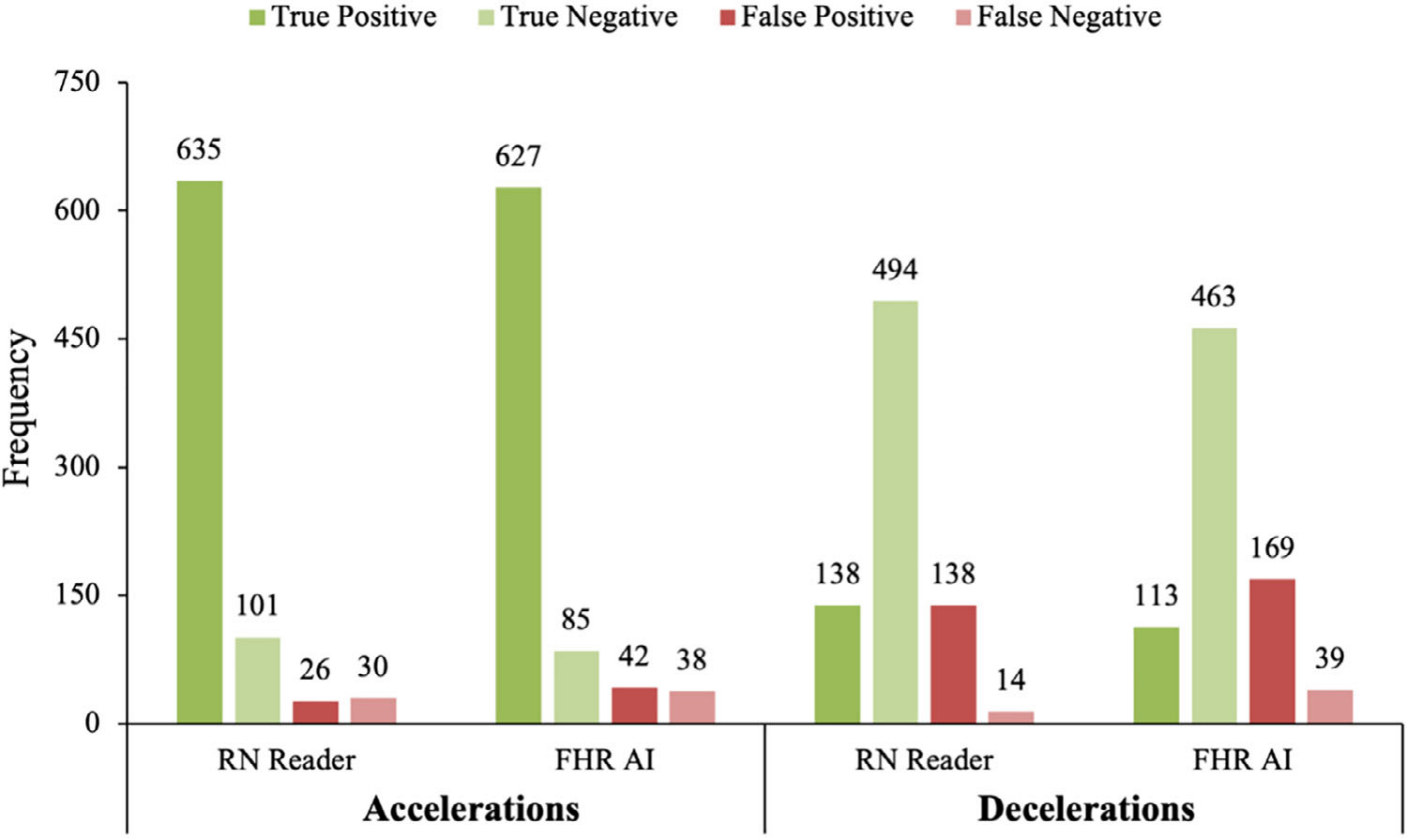
Frequency of true and false positive/negatives for accelerations and decelerations. For both RN reader and AI algorithm assessments of accelerations and decelerations, outcomes are categorized by frequency of true positives (dark green), true negatives (light green), false positives (dark red), and false negatives (light red). AI, artificial intelligence; FHR, fetal heart rate; RN, registered nurse.

The algorithm performance was evaluated using the AR determined by the panel (Table 3). For the primary endpoints examining software device functions, the FHR AI algorithm showed comparable results to the RN reader, with percent differences (95% CI) in AR (RN reader–AI) of 3.38% (−1.5%, 8.25%) for baseline value, 3.03% (0.72%, 5.34%) for accelerations, 7.14% (3.68%, 10.61%) for decelerations, 8.25% (3.57%, 12.93%) for contraction frequency, and 22.13% (17.39%, 26.86%) for contraction duration. The FHR AI algorithm also performed comparably to RN readers for the secondary endpoints of the derived functions: variability and deceleration type. The difference in ARs between RN readers and the algorithm was −1.75% (95% CI, −4.10%, 0.60%) for variability and 9.13% (95% CI, 5.38%, 12.88%) for deceleration type. Exploratory endpoints of sensitivity and specificity, calculated through true and false positives/negatives (Figure 3), were similar between the FHR AI algorithm and RN readers (Table 4). Subgroup exploratory analyses for the primary and secondary endpoints by race and ethnicity, BMI, and site are provided in Tables S1–S3.

**TABLE 3:** Acceptance rate comparison between RN reader and FHR AI algorithm.

	RN reader	FHR AI	Difference	*p* value
Primary endpoints (software device functions)				
Baseline	69.25% (66.05%, 72.45%)	65.88% (62.59%, 69.16%)	3.38% (−1.50%, 8.25%)	<0.0001
Accelerations^a^	92.93% (91.14%, 94.71%)	89.90% (87.80%, 92.00%)	3.03% (0.72%, 5.34%)	<0.0001
Decelerations^b^	80.61% (77.84%, 83.38%)	73.47% (70.38%, 76.56%)	7.14% (3.68%, 10.61%)	<0.0001
Contraction Frequency	43.00% (39.57%, 46.43%)	34.75% (31.45%, 38.05%)	8.25% (3.57%, 12.93%)	0.0023
Contraction Duration	44.00% (40.56%, 47.44%)	21.87% (19.01%, 24.74%)	22.13% (17.39%, 26.86%)	0.9984
Secondary endpoints (derived functions)				
Variability	91.38% (89.43%, 93.32%)	93.13% (91.37%, 94.88%)	−1.75% (−4.10%, 0.60%)	<0.0001
Deceleration Type	74.00% (70.96%, 77.04%)	64.88% (61.57%, 68.18%)	9.13% (5.38%, 12.88%)	0.0011

*Note*: Significance indicates non‐inferiority of the algorithm (i.e., null hypothesis: H_0_: [AR_rn reader_] − [AR_algorithm_] ≥ 0.15).

Abbreviations: AI, artificial intelligence; FHR, fetal heart rate; RN, registered nurse.

^a^ Cohort size: *n* = 792.

^b^ Cohort size: *n* = 784.

**TABLE 4:** Sensitivity and specificity of accelerations and decelerations.

	RN reader	FHR AI
Accelerations		
Sensitivity	0.9549 (0.9391, 0.9707)	0.9429 (0.9252, 0.9605)
Specificity	0.7953 (0.7251, 0.8655)	0.6693 (0.5875, 0.7511)
Decelerations		
Sensitivity	0.9079 (0.8619, 0.9539)	0.7434 (0.6740, 0.8129)
Specificity	0.7816 (0.7474, 0.8139)	0.7326 (0.6981, 0.7671)

*Note*: Values presented in parentheses represent 95% CI.

Abbreviations: AI, artificial intelligence; CI, confidence interval; FHR, fetal heart rate; RN, registered nurse.

An analysis further examining contraction duration was conducted to assess how the difference in AR between RN readers and the AI algorithm was affected by allowing for 20 s of variability in contraction duration. Following these adjustments, AR decreased from 22.13% (17.39%, 26.86%) in the original approach to 13.75% (9.89%, 17.61%; Table 5).

**TABLE 5:** Additional analysis of contraction duration.

	No adjustment (original results)	With adjustment to allow 20 s variability
Panel acceptances		
FHR AI	118	77
RN reader	295	187
Both	57	206
None	330	330
Total	800	800
Acceptance rates		
RN reader acceptance rate	44.00% (40.56%, 47.44%)	49.13% (45.66%, 52.59%)
FHR AI acceptance rate	21.87% (19.01%, 24.74%)	35.38% (32.06%, 38.69%)
Acceptance rate difference (RN reader − FHR AI)	22.13% (17.39%, 26.86%)	13.75% (9.89%, 17.61%)

*Note*: Values presented in parentheses represent 95% CI.

Abbreviations: AI, artificial intelligence; FHR, fetal heart rate; RN, registered nurse.

Furthermore, in the second post hoc analysis evaluating the frequency distribution of differences in paired contraction duration values, 85.11% of the independent paired observations fell within the 20‐s variance threshold. The difference in contraction duration between the RN readers and the FHR AI was not significant, with a mean difference of −0.66 s and a median of 0 s.

## 4 DISCUSSION

This study presents a non‐inferiority analysis comparing a novel FHR AI algorithm with RN reader assessment of CTG tracings. Algorithm non‐inferiority was established for software device function primary endpoints of baseline, accelerations, decelerations, and contraction frequency, and the secondary endpoints of derived functions of variability and deceleration type. The algorithm did not meet the predetermined non‐inferiority endpoint for contraction duration, but considerable improvement was observed after adjusting the time‐range variability. These results suggest that the FHR AI algorithm may be developed to become a valuable clinical decision support tool. By providing consistent, real‐time, objective assessments during labor, the software may reduce the inter‐ and intra‐observer variability associated with manual tracing assessment. Notably, the AI algorithm provides a software analysis intended to support, rather than replace, human bedside decision‐making and safety interventions.

In accordance with the NICHD guidelines [3], the AI algorithm output is designed to be interpretable across varying levels of expertise. This study demonstrated that the RN readers and algorithm labeling were similar, as measured by the AR from the expert panel. The AR results suggest that clinical implementation of algorithm outputs may readily support existing bedside clinical practice. The use of future algorithms may simplify clinician workload, reduce repetitive manual tasks, and enhance efficiency; all of which may address clinical burnout. Steps taken to reduce burnout today result in alleviated workforce shortages and reduced costs in the future [10].

All primary and secondary endpoints achieved non‐inferiority with the exception of contraction duration. Contraction duration had a 22.13% (17.39%, 26.86%) inferiority difference between RN readers and the algorithm, which was higher than the 15% non‐inferiority threshold. This can partially be explained by inconsistencies in the assessment of contraction duration, particularly due to challenges in visually assessing the beginning or end of a contraction and a lack of well‐defined criteria. It is possible there is an increased sensitivity of the AI algorithm in its ability to detect small changes in UA versus the baseline. These small changes may not be consistently identified by the RN reader due to the limitations of human visual resolution, or they may not be perceived as a clinically relevant element of the contraction. When a repeat analysis of contraction duration was conducted with the introduction of 20 s of allowed duration assessment variability, AR improved to 13.75% (9.89%, 17.61%), with the upper bound slightly exceeding the 15% non‐inferiority margin. Whether the 20‐s variability is consistent with intra‐ and inter‐clinician differences in interpretation is not known. Accuracy requirements for the assessment of contraction duration have also not been established. Much of labor contraction management involves identifying patterns and frequency of contractions rather than specific durations, with some conditions, such as tachysystole or uterine tetany, representing exceptions requiring prompt intervention.

During the development, training, and validation of the FHR AI algorithm, adherence to the foundational principles of responsible AI, aligned with characteristics defined by the National Institute of Standards and Technology's Artificial Intelligence Risk Management Framework [11], was maintained. When developing and evaluating AI in healthcare, professionals must remain vigilant about its validity and reliability, as well as the underlying assumptions and potential sources of error inherent in AI and machine learning [10]. To promote ethical deployment and development of the FHR AI algorithm, a multi‐disciplinary team of scientists, health informatics personnel, and healthcare professionals provided oversight, emphasizing data privacy, bias mitigation, and diverse patient representation [8]. Incorporating principles of responsible AI in the development and validation of this novel FHR AI algorithm was essential to ensure its ethical use, transparency, and fairness, ultimately fostering trust and reliability in clinical decision‐making.

Patient tracings in this study were collected from patients of diverse demographic backgrounds, spanning different races and ethnicities, ages, and stages of pregnancy, to ensure the algorithm's generalizability to a diverse intended population. It is of particular importance that future investigations ensure algorithms can perform safely and effectively in populations of women disproportionately affected by discrimination, systemic bias, and social injustice in the healthcare field, which are frequently associated with poorer maternal outcomes, and evaluate their potential to mitigate inherent clinician biases [12]. In addition to race and ethnic diversity, this study included women with a wide range of maternal ages, <20 years to >40 years, to include those who are at increased risk for complications due to physiological, hormonal, and social factors [12]. The commitment to incorporate tracings of diverse demographics during both the algorithm's development, which utilized a final training set of 1600 highly curated tracings drawn from an initial pool of 222,000 maternity files across 19 birthing centers, and the current study reduces the probability of algorithmic bias that may stem from skewed training datasets [8]. While subgroup exploratory analyses are provided in the supplement, this study was solely powered for the general population (*n* = 731), and some subgroup sample sizes were as small as 6 and 16 patients for BMI and site, respectively; therefore, any subgroup variance may reflect underpowered sample sizes, and hypothesis testing cannot be done.

Real‐world studies are warranted to capture the potential benefits of the use of this decision‐support tool in diverse clinical settings and populations [10]. Documentation of algorithm usage with linkage to patient health records will be essential to assess the impact on health equity and maternal‐fetal outcomes. As the use of AI‐powered tools is projected to increase extensively in healthcare [13], dedicated procedure codes to record its use during care and support the conduct of effective future clinical research may be beneficial.

This study presents several limitations. First, the non‐inferiority margin of 15% may be considered wide and could mask small yet clinically significant differences. Second, the foundation of this study assumes that RN assessment of CTG tracings represents the ground truth; however, interobserver variability in clinical practice may impact the reliability of this comparator, particularly given the varying roles of RNs and other healthcare professionals. This research serves as a preliminary effort to mitigate clinical subjectivity through AI, and the automated assessment of baseline elements introduced here represents the first step toward this goal and eventual risk prediction. Third, exploratory endpoints revealed that the FHR AI algorithm demonstrated a lower sensitivity in detecting decelerations compared to RN readers. Because accurate deceleration identification is a critical step in recognizing tracings that may require prompt intervention, this further reinforces that the algorithm is designed to assist healthcare professionals at the bedside, who remain responsible for synthesizing AI outputs and making clinical decisions. Lastly, the study was conducted in a controlled academic setting, which may not fully replicate the variability of real‐world clinical environments. Future real‐world studies are recommended to accurately capture the algorithm's impact amid everyday clinical conditions, such as workflow challenges, high‐stress environments, cost‐effectiveness, and maternal‐fetal outcomes.

## 5 CONCLUSION

This study demonstrated the non‐inferiority of a novel FHR AI algorithm for analyzing and assessing FHR and UA data compared with RN readers. These findings underscore the potential of AI algorithms to support clinical decision‐making and reduce variability in obstetric care. By providing analyses consistent with those of RNs, FHR AI algorithms may reduce interobserver variability and help clinicians assess fetal parameters with greater confidence. Future prospective studies using neonatal endpoints will be essential to determine if such tools can influence real‐world clinical decision‐making, alleviate clinician workload, and improve patient outcomes.

## CONFLICT OF INTEREST STATEMENT

R.P., R.V., S.H., K.B., and J.W.B. are employees of GE HealthCare. H.O.Y. is an employee of Boston Strategic Partners, which was supported by GE HealthCare for this study.

## ETHICS STATEMENT

This retrospective study was determined exempt from Institutional Review Board (IRB) oversight since the research and analysis were limited to data gathered initially for non‐research purposes, adhering to privacy laws and IRB exemption criteria 45CFR46.104(d)(4). In accordance with national legislation and institutional requirements, informed consent was not required for the use of this de‐identified, retrospectively collected data.

## Supporting information



Supporting Information

## Data Availability

Due to the legal/commercial nature of the research, supporting data are not available.
