# Supplementary material for: Non‐inferiority analysis of a fetal heart rate artificial intelligence algorithm to registered nurse assessment
Source: Pregnancy (Hoboken). 2026 Aug 27;2(5):e70433. doi: 10.1002/pmf2.70433 (PMC13520920; doi:10.1002/pmf2.70433)
Supplement: Supplementary file 1 [file PMF2-2-e70433-s001.docx]

**Supplemental Table 1.** Primary and Secondary Endpoints Race and Ethnicity Subgroup Exploratory Analysis

|  | **RN – AI AR Difference**  **(95% CI)** | **Lower 95% CI** | **Upper 95% CI** |
| --- | --- | --- | --- |
| **Baseline** |  | | |
| Non-Hispanic White | 5.62% | -1.25% | 12.50% |
| Non-Hispanic Black | 5.38% | -6.35% | 17.12% |
| Hispanic | 5.13% | -7.81% | 18.07% |
| Non-Hispanic Asian | -12.12% | -28.12% | 3.88% |
| Other Race or Ethnicity | 0.00% | -15.90% | 15.90% |
| Missing | - | - | - |
| **Variability** |  | | |
| Non-Hispanic White | 0.00% | -3.03% | 3.03% |
| Non-Hispanic Black | -1.54% | -7.56% | 4.49% |
| Hispanic | -5.98% | -11.92% | -0.04% |
| Non-Hispanic Asian | -3.03% | -12.39% | 6.33% |
| Other Race or Ethnicity | -2.63% | -11.55% | 6.28% |
| Missing | - | - | - |
| **Acceleration** |  | | |
| Non-Hispanic White | 2.22% | -0.71% | 5.15% |
| Non-Hispanic Black | 9.30% | 2.36% | 16.25% |
| Hispanic | -1.71% | -7.97% | 4.55% |
| Non-Hispanic Asian | 1.56% | -6.53% | 9.66% |
| Other Race or Ethnicity | 6.67% | -0.08% | 13.41% |
| Missing | - | - | - |
| **Deceleration** |  | | |
| Non-Hispanic White | 5.06% | 0.38% | 9.74% |
| Non-Hispanic Black | 10.94% | 1.69% | 20.18% |
| Hispanic | 6.84% | -2.25% | 15.93% |
| Non-Hispanic Asian | 7.58% | -2.10% | 17.25% |
| Other Race or Ethnicity | 11.84% | -1.29% | 24.98% |
| Missing | - | - | - |
| **Decel Type** |  | | |
| Non-Hispanic White | 7.6% | 2.7% | 12.5% |
| Non-Hispanic Black | 16.9% | 7.1% | 26.7% |
| Hispanic | 6.8% | -3.4% | 17.1% |
| Non-Hispanic Asian | 10.6% | -0.6% | 21.8% |
| Other Race or Ethnicity | 6.6% | -8.6% | 21.8% |
| Missing | - | - | - |
| **Contraction Frequency** |  | | |
| Non-Hispanic White | 10.76% | 4.31% | 17.21% |
| Non-Hispanic Black | 10.00% | -1.84% | 21.84% |
| Hispanic | 2.56% | -8.91% | 14.04% |
| Non-Hispanic Asian | -4.55% | -22.58% | 13.48% |
| Other Race or Ethnicity | 11.84% | -3.18% | 26.86% |
| Missing | 0.00% | 0.00% | 0.00% |
| **Contraction Duration** |  | | |
| Non-Hispanic White | 21.5% | 14.9% | 28.1% |
| Non-Hispanic Black | 22.3% | 9.8% | 34.8% |
| Hispanic | 20.5% | 9.0% | 32.0% |
| Non-Hispanic Asian | 21.2% | 3.6% | 38.8% |
| Other Race or Ethnicity | 26.3% | 12.0% | 40.6% |
| Missing | - | - | - |

**Supplemental Table 2.** Primary and Secondary Endpoints BMI Subgroup Exploratory Analysis

|  | **RN – AI AR Difference**  **(95% CI)** | **Lower 95% CI** | **Upper 95% CI** |
| --- | --- | --- | --- |
| **Baseline** |  | | |
| Underweight | 16.67% | -38.32% | 71.65% |
| Normal | 3.05% | -8.09% | 14.19% |
| Overweight | -3.59% | -12.62% | 5.45% |
| Obese | 8.00% | 0.92% | 15.08% |
| Missing | -3.13% | -26.82% | 20.57% |
| **Variability** |  | | |
| Underweight* | 16.67% | -13.15% | 46.49% |
| Normal | 0.00% | -5.07% | 5.07% |
| Overweight | 1.35% | -3.38% | 6.08% |
| Obese | -2.67% | -5.79% | 0.46% |
| Missing | -10.00% | -25.00% | -40.00% |
| **Acceleration** |  | | |
| Underweight* | 0.00% | 0.00% | 0.00% |
| Normal | 4.27% | -0.62% | 9.15% |
| Overweight | 4.09% | -0.15% | 8.33% |
| Obese | 1.62% | -1.88% | 5.12% |
| Missing | 6.45% | -5.99% | 18.89% |
| **Deceleration** |  | | |
| Underweight* | 13.15% | -16.67% | -46.49% |
| Normal | 10.00% | 2.21% | 17.79% |
| Overweight | 4.98% | -2.03% | 11.99% |
| Obese | 7.63% | 2.68% | 12.58% |
| Missing | 6.67% | -2.26% | 15.59% |
| **Decel Type** |  | | |
| Underweight | 0.0% | -46.2% | 46.2% |
| Normal | 9.1% | 0.9% | 17.4% |
| Overweight | 7.6% | -0.2% | 15.5% |
| Obese | 10.9% | 5.7% | 16.1% |
| Missing | 0.0% | -15.0% | 15.0% |
| **Contraction Frequency** |  | | |
| Underweight | 33.33% | -26.31% | 92.97% |
| Normal | 14.63% | 4.18% | 25.09% |
| Overweight | 8.07% | -0.83% | 16.97% |
| Obese | 5.33% | -1.46% | 12.13% |
| Missing | 6.25% | -14.86% | 27.36% |
| **Contraction Duration** |  | | |
| Underweight | 0.0% | -65.3% | 65.3% |
| Normal | 22.6% | 11.6% | 33.6% |
| Overweight | 19.3% | 10.4% | 28.2% |
| Obese | 21.9% | 15.1% | 28.7% |
| Missing | 46.9% | 27.5% | 66.2% |

Note: Estimates from the underweight subcategory do not have enough sample size to make meaningful conclusions.

* CI calculated using Mantel-Haenszel method in procedure frequency

**Supplemental Table 3.** Primary and Secondary Endpoints Site Subgroup Exploratory Analysis

|  | **RN – AI AR Difference**  **(95% CI)** | **Lower 95% CI** | **Upper 95% CI** |
| --- | --- | --- | --- |
| **Baseline** |  | | |
| 1 | 2.41% | -4.00% | 8.83% |
| 2 | 4.04% | -5.62% | 13.69% |
| 3 | -5.33% | -20.52% | 9.86% |
| 4 | 16.67% | -6.12% | 39.46% |
| 5 | 37.50% | 13.78% | 61.22% |
| **Variability** |  | | |
| 1 | -0.44% | -3.65% | 2.78% |
| 2 | -4.04% | -8.22% | 0.15% |
| 3 | -5.33% | -12.63% | 1.96% |
| 4 | 3.33% | -7.92% | 14.59% |
| 5 | 0.00% | -17.32% | 17.32% |
| **Acceleration** |  | | |
| 1 | 3.10% | -0.14% | 6.34% |
| 2 | 0.45% | -3.59% | 4.50% |
| 3 | 8.00% | 0.83% | 15.17% |
| 4 * | 10.00% | -0.74% | 20.74% |
| 5 | 0.00% | 0.00% | 0.00% |
| **Deceleration** |  | | |
| 1 | 4.92% | 0.47% | 9.37% |
| 2 | 13.51% | 6.68% | 20.35% |
| 3 | 6.67% | -5.21% | 18.55% |
| 4 | 0.00% | -18.47% | 18.47% |
| 5 | -7.14% | -20.63% | 6.35% |
| **Decel Type** |  | | |
| 1 | 8.1% | 3.2% | 13.1% |
| 2 | 14.8% | 7.4% | 22.2% |
| 3 | 5.3% | -7.4% | 18.1% |
| 4 | -3.3% | -17.9% | 11.2% |
| 5 | 0.0% | -17.3% | 17.3% |
| **Contraction Frequency** |  | | |
| 1 | 7.02% | 0.62% | 13.42% |
| 2 | 11.21% | 2.68% | 19.74% |
| 3 | 5.33% | -9.86% | 20.52% |
| 4 | 6.67% | -13.85% | 27.19% |
| 5 | 18.75% | -7.05% | 44.55% |
| **Contraction Duration** |  | | |
| 1 | 18.0% | 11.5% | 24.5% |
| 2 | 27.8% | 19.2% | 36.4% |
| 3 | 30.7% | 16.9% | 44.5% |
| 4 | 16.7% | -7.9% | 41.3% |
| 5 | 31.3% | 2.7% | 59.8% |

Note: Estimates from the Site 5 subcategory do not have enough sample size to make meaningful conclusions.

* CI calculated using Mantel-Haenszel method in procedure frequency
